# Clinical predictors of dengue fever co-infected with leptospirosis among patients admitted for dengue fever – a pilot study

**DOI:** 10.1186/s12929-017-0344-x

**Published:** 2017-06-28

**Authors:** Jeyanthi Suppiah, Shie-Yien Chan, Min-Wern Ng, Yam-Sim Khaw, Siew-Mooi Ching, Lailatul Akmar Mat-Nor, Naematul Ain Ahmad-Najimudin, Hui-Yee Chee

**Affiliations:** 1 Department of Medical Microbiology and Parasitology, Faculty of Medicine and Health Sciences, Universiti Putra Malaysia, Selangor, Malaysia; 2 Virology Unit, Infectious Disease Research Centre, Institute for Medical Research, Kuala Lumpur, Malaysia; 3 Department of Family Medicine, Faculty of Medicine and Health Sciences, Universiti Putra Malaysia, Selangor, Malaysia; 4 Malaysian Research Institute on Ageing, Faculty of Medicine and Health Sciences, Universiti Putra Malaysia , Selangor, Malaysia; 5 Microbiology Unit, Department of Pathology, Hospital Serdang, Selangor, Malaysia

**Keywords:** Dengue fever, Leptospirosis, Co-infection, Shock, Serotype, Malaysia

## Abstract

**Background:**

Dengue and leptospirosis infections are currently two major endemics in Malaysia. Owing to the overlapping clinical symptoms between both the diseases, frequent misdiagnosis and confusion of treatment occurs. As a solution, the present work initiated a pilot study to investigate the incidence related to co-infection of leptospirosis among dengue patients. This enables the identification of more parameters to predict the occurrence of co-infection.

**Method:**

Two hundred sixty eight serum specimens collected from patients that were diagnosed for dengue fever were confirmed for dengue virus serotyping by real-time polymerase chain reaction. Clinical, laboratory and demographic data were extracted from the hospital database to identify patients with confirmed leptospirosis infection among the dengue patients. Thus, frequency of co-infection was calculated and association of the dataset with dengue-leptospirosis co-infection was statistically determined.

**Results:**

The frequency of dengue co-infection with leptospirosis was 4.1%. Male has higher preponderance of developing the co-infection and end result of shock as clinical symptom is more likely present among co-infected cases. It is also noteworthy that, DENV 1 is the common dengue serotype among all cases identified as dengue-leptospirosis co-infection in this study.

**Conclusion:**

The increasing incidence of leptospirosis among dengue infected patients has posed the need to precisely identify the presence of co-infection for the betterment of treatment without mistakenly ruling out either one of them. Thus, anticipating the possible clinical symptoms and laboratory results of dengue-leptospirosis co-infection is essential.

## Background

Dengue fever and leptospirosis infections have emerged as an important concern of public health worldwide, particularly in tropical and subtropical regions. Dengue fever is an arthropod born viral disease [1] whereas leptospirosis is a zoonotic disease caused by the spirochetes of the genus *Leptospira* [2].

Often, dengue and leptospirosis are diagnosed separately. The gold standard test to identify leptospira is the microscopic agglutination test (MAT) while IgM antibody detection by using enzyme-linked immunosorbent assay (ELISA) is also widely used nowadays [3]. Further, the confirmatory test for dengue is known as dengue combo test which includes the detection of dengue immunoglobulin M (IgM) and immunoglobulin G (IgG) serology with non-structural 1 (NS1) antigen [4]. Additionally, molecular diagnosis by using the Real-Time Polymerase Chain Reaction (Rea-Time PCR) is also performed concurrently with serology for confirmation and detection of dengue virus.

In the recent years, co-infections of dengue cases with leptospirosis have been reported with frequency ranging from 0.9-8% [5–7]. Both the infections generally present with a phase of acute febrile illness which includes the sudden onset of fever, headache, and myalgia [8]. Due to the overlapping clinical presentations between these two diseases, ability to diagnose them became more challenging especially with the presence of acute co-infection. It is very crucial to be able to distinguish leptospirosis from dengue as early commencement of antibiotic therapy in cases with leptospirosis leads to a more favorable outcome, while dengue can be treated symptomatically [9].

Therefore, we designed a retrospective study, aiming to screen the patients that were admitted for dengue fever at Hospital Serdang and to determine the prevalence of co-infection with leptospirosis. Other than that, this study also attempts to identify the clinical predictors of both the co-infection.

## Methods

### Study subject

In this study, serum specimens were obtained from a total of 268 randomly selected patients that were admitted primarily for dengue fever at Hospital Serdang, Malaysia in between 2014-2016. Clinical, demographic and laboratory data were retrieved from the patients’ database available in the hospital. Besides, leptospirosis laboratory testing (performed by hospital) were also recorded for those dengue patients who were also suspected for leptospirosis infection.

### Dengue viral RNA extraction

Dengue viral ribonucleic acid (RNA) was extracted from the serum specimens by using the QIAamp Viral RNA Mini Kit (Qiagen) following the manufacturer’s instructions. During the final procedure of the spin column based method, 50 μl of viral RNA was eluted from each serum specimen. This were then used as template for the one-step Real-Time PCR amplification.

### Serotyping by Multiplex Real-Time PCR

Subsequently, extracted dengue viral RNAs were subjected for serotyping by using the commercially available abTES DEN 4 qPCR I kit (AITbiotech). The PCR reactions are composed of 12.5 µl of 2X RT (reverse transcriptase)-PCR Mix, 1.5 μl of Primer/Probe Mix, 0.5 μl of RT/Taq Enzyme Mix, 0.1 μl of Internal Control, 5.0 μl of RNA template and 5.4 μl of nuclease-free water. As for the negative control, the RNA template was replaced with 5 μl of nuclease-free water. Real-Time PCR amplification was performed on the CFX 96 (Biorad) platform with the cycling condition recommended by the manufacturer.

### Data analysis

The study subjects were categorized into two groups, namely dengue-leptospirosis co-infection and dengue infection without leptospirosis. Statistical analysis was done by using the IBM statistical package for the social sciences (SPSS) version 21.0. Categorical data were reported in terms of proportions (percentage). Chi-square test was used for the categorical variables. Further, multiple logistic regression analysis was used to identify the clinical predictors of dengue-leptospirosis co-infection. Variables with the p value less than 0.25 in univariate analysis square test as well as all other clinically significant variables were entered into the multiple logistic regressions. The dependent variable was dengue-leptospirosis co-infection while the selected independent variables for multiple logistic regressions were gender, shock, lethargy and abdominal pain. All analyses were made with 95% confidence interval (CI), and the level of significance was set at *p* < 0.05.

## Results

### *Dengue virus serotypes and prevalence of* dengue-leptospirosis co-infection

The Real-Time PCR assay detects the dengue virus based on the four fluorescence channels and one additional channel for internal control. The channels that were used are Cy5 (DENV 1), FAM (DENV 2), Texas red (DENV 3), Quasar 705 (DENV 4) and HEX for the internal control. From the total of 268 dengue fever cases that were included in this study, the prevalence of dengue serotypes were DENV 1 (67.5%), DENV 2 (15.7%), DENV 3 (6.3%), dual infections of DENV 1/2 (7.1%), DENV 1/3 (1.9%), DENV 2/3 (1.1%) and DENV 2/4 (0.4%). For those dengue patients that were suspected for leptospirosis, the hospital proceeded with further test, the IgM assay. Additionally, the reference laboratory conducted the MAT (Table 1). Based on the data obtained from the laboratory investigation, 11 (4.1%) dengue cases out of the total 268 cases were found to be co-infected with leptospirosis. Although no significant association were found between the dengue serotypes and the leptospirosis co-infection, it is noteworthy that all the 11 co-infected cases belongs to the serotype DENV 1.

**Table 1:** Serology data for dengue cases suspected for leptospirosis

Total dengue-lepto suspected cases	IgM assay (latex agglutination)		MAT assay		
n = 11	neg (%,n)	pos (%, n)	neg (%,n)	pos (%, n)	inconclusive (%,n)
	0 (0)	100 (11)	18.2 (2) Titer < 50	27.3 (3) Titer ≥ 400	54.5 (6) Titer ≥ 50 - < 400

### Association of age and gender with dengue-leptospirosis co-infection

The association between the demographic data such as age and gender among cases of dengue fever with leptospirosis and non-leptospirosis groups among the study population was analyzed. There was no significant association found between age and the two groups with the mean of 32.3 ± 9.4 and 30.8 ± 10.8 respectively. Besides that, gender was proven to be significantly associated with the outcome of co-infection whereby male patients were more likely to develop dengue fever with leptospirosis compared to female patients (*P* = 0.03).

### Association of clinical symptoms with dengue-leptospirosis co-infection

The association between the various spectrums of clinical symptoms and presentation with dengue-leptospirosis co-infection was investigated and tabulated in Table 2. The outcome of the analysis showed that shock was a significant symptoms that was shared in common by patients presented with dengue-leptospirosis co-infection (*P* = 0.001). Other than that, vomiting was seen more often among the cases of dengue fever alone rather than dengue with leptospirosis co-infection. As well, symptoms such as headache, arthralgia, epigastric pain, retro-orbital pain, acute kidney injury, gum bleeding, hepatitis and bleeding tendency were predominantly seen among the cases of dengue fever alone while absent in the dengue-leptospirosis group. On top of that, it was also found that frequency of abdominal pain and lethargy were higher and more specific to the dengue-leptospirosis group. Also, a minor difference was seen in terms of number of days of onset of fever between the co-infection and non-co-infection groups with an average of 4.2 ± 2.8 and 4.7 ± 1.8 respectively. Further, the dengue infection phases, namely the febrile, critical and recovery phases were more precisely observed and seen among the cases of dengue fever without co-infection with leptospirosis. Although the frequency of defervescence phase was higher in the dengue-leptospirosis group, it was statistically insignificant. Remarkably, no severe dengue cases were recorded among the group of dengue co-infected with leptospirosis.

**Table 2:** Association of clinical symptoms relative to leptospirosis dengue co-infection

Patient characteristics		Dengue-leptospirosis co-infection (*n* = 11)	Dengue infectionwithout leptospirosis (*n* = 257)	*P*-value*
Age	mean (years)	32.3 ± 9.4	30.8 ± 10.8	0.65
Day of fever onset	mean (days)	4.2 ± 2.8	4.7 ± 1.8	0.48
Gender	Male (%, n)	90.9(10)	57.6(148)	0.03*
	Female (%, n)	9.1(1)	42.4(109)	
Warning sign	Yes (%, n)	36.4(4)	39.7(102)	0.83
	No (%, n)	63.6(7)	60.3(155)	
Headache	Yes (%, n)	0(0)	4.3(11)	0.48
	No (%, n)	63.6(7)	95.7(246)	
Dengue infection phase	febrile	72.0(8)	74.7(192)	0.88
	defervescence	27.3(3)	21.0(54)	
	critical	0(0)	1.6(4)	
	recovery	0(0)	2.7(7)	
Severe dengue	Yes (%, n)	0(0)	1.2(3)	0.72
	No (%, n)	100(11)	98.8(254)	
Arthralgia	Yes (%, n)	0(0)	6.6(17)	0.38
	No (%, n)	100(11)	93.4(240)	
Vomiting	Yes (%, n)	9.1(1)	15.6(40)	0.56
	No (%, n)	90.9(10)	84.4(217)	
Epigastric pain	Yes (%, n)	0(0)	7.8(20)	0.34
	No (%, n)	100(11)	92.2(237)	
Abdominal pain	Yes (%, n)	9.1(1)	2.3(6)	0.17*
	No (%, n)	90.9(10)	97.7(251)	
Retro-orbital pain	Yes (%, n)	0(0)	1.2(3)	0.72
	No (%, n)	100(11)	98.8(254)	
Lethargy	Yes (%, n)	9.1(1)	2.7(7)	0.22*
	No (%, n)	90.0(10)	97.3(250)	
Shock	Yes (%, n)	9.1(1)	0.4(1)	0.001*
	No (%, n)	90.9(10)	99.6(256)	
Acute kidney injury	Yes (%, n)	0(0)	0.4(1)	0.84
	No (%, n)	100(11)	99.6(256)	
Gum bleeding	Yes (%, n)	0(0)	2.3 (6)	0.61
	No (%, n)	100(11)	97.7(251)	
Bleeding tendency	Yes (%, n)	0(0)	3.5(9)	0.53
	No (%, n)	100(11)	96.5(248)	
Hepatitis	Yes (%, n)	0(0)	11.7(30)	0.23
	No (%, n)	100(11)	88.3(227)	

Bold numbers represent significant *P* value (<0.05)

* Indicates variables with *P* value <0.25 which were selected for the mutiple logistic regression analysis

### Association of laboratory results with dengue-leptospirosis co-infection

The laboratory parameters such as level of aspartate transaminase (AST), alanine transaminase (ALT), platelet count, haematocrit and creatine phosphokinase (CPK) levels were analyzed to identify the possible predictors of dengue-leptospirosis co-infection (Table 3). However, there was no significant association found between these parameters and the dengue-leptospirosis co-infection.

**Table 3:** Association of laboratory results with dengue- leptospirosis co-infection

Laboratory results		Leptospirosis dengue (*n* = 11)	Not leptospirosis-dengue infection (*n* = 257)	*P*-value*
Type of dengue serotype	DENV 1	100(11)	66.1(170)	0.49
	DENV 2	0(0)	16.3(42)	
	DENV 3	0(0)	6.6(17)	
	DENV 1 + DENV 2	0(0)	7.4(19)	
	DENV 1 + DENV 3	0(0)	1.9(5)	
	DENV 2 + DENV 3	0(0)	1.2(3)	
	DENV 2 + DENV 4	0(0)	0.4(1)	
Haematocrit ≥20% increase from baseline	Yes (%, n)	18.2(2)	8.6(22)	0.47
	No (%, n)	81.8(9)	91.4(235)	
Platelet count <100 x 10^9^ /L)	Yes (%, n)	18.2(2)	10.5(27)	0.42
	No (%, n)	81.8(9)	89.5(230)	
ALT	Mean (mmol/L)	122.2 ± 88.8	98.33 ± 116.2	0.50
AST	Mean (mmol/L)	134.64 ± 81.4	127.39 ± 142.8	0.87
CPK	Mean (U/L)	1355.3 ± 1765.4	484.0 ± 1453.638	0.06*

* Indicates variables with *P* value <0.25 which were selected for the mutiple logistic regression analysis

### Predictors of dengue-leptospirosis co-infection

Multiple logistic regression analysis was used to identify the predictors of dengue-leptospirosis co-infection among the statistically significant parameters and selected parameters with the *P* value of <0.25 from the chi-square analysis (Table 2). It was found that the symptoms of shock has significant predictive value for dengue-leptospirosis co-infection (Table 4).

**Table 4:** Multiple logistic based regression analysis for predictors of dengue-leptospirosis co-infection

Clinical variables	Exp (B)	95% CI		*P*-value
		Lower	Upper	
Shock: Presence	**39.17**	**1.32**	**1161.32**	**0.03**
Absence	1.00			
Sex: Male	**8.86**	**0.96**	**81.81**	**0.05**
Female	1.00			
Abdominal pain: Presence	2.92	0.55	15.47	0.21
Absence	1.00			
Lethargy: Presence	5.34	0.50	56.53	0.16
Absence	1.00			
CPK	1.00	1.00	1.00	0.18

Bold numbers represent significant *P* value (<0.05)

## Discussion

The study revealed the distribution of the dengue serotype among patients admitted to Hospital Serdang in between 2014-2016 and further analyzed the association between demographic, clinical and laboratory parameters with dengue-leptospirosis co-infection. During the period of study, it was found that the DENV 1 was the most predominant serotype seen in circulation. However, the circulating dengue serotype pattern in Malaysia has always been inconsistent and varies among states. Into details, in 2013, Malaysia experienced an unprecedented dengue outbreak which continued to peak in the year 2014 and 2015. Indescribably, from March 2013 onwards, it was reported that DENV 2 overtook a pre-existing DENV 4 dominance pattern [10, 11]. A surveillance study conducted by Ministry of Health Malaysia demonstrated that DENV 1 have replaced DENV 2 twice in 2014 (March and June-December) [11]. Further, it has been observed that shift in the dengue serotype often causes surge in the number of cases owing to the reduced herd immunity to the new serotype. A study conducted at Klang Valley postulated that DENV 1 virus has a cycling pattern of outbreak and suggested the possibility that it would be selected to be the clade replacement candidate initiating the next outbreak [12].

Other than that, in the present study, the frequency of co-infection with leptospirosis was found to be 4.1%. To our best knowledge, this is a pioneer study conducted to investigate the incidence rate of dengue-leptospirosis co-infection in Malaysia by confirmed laboratory tests. Other studies in Malaysia have reported the prevalence of either dengue infection or leptospirosis infection and leptospirosis infection among dengue negative patients. The incidence of co-infection has also been documented in other countries such as India, Barbados, Mexico, Sri-Lanka, Jamaica and Brazil ranging from the year 2000-2015 [6–8, 13–21]. Of these studies, many are merely individual case–report based and only a countable number have reported concomitant leptospirosis and dengue infection in a specific population similar to our study. The findings from our study is important as Malaysia is highly endemic for both dengue and leptospirosis, yet no studies have attempted to investigate such co-occurrence.

The fundamental reason related to this co-infection was often referred as random and related to the climate, because the both diseases peaked during either monsoon season or heavy rainfall. However, dengue and leptospirosis co-existence need to be confirmed whether it is selective due to some synergetic reactions or completely random. Certain literature in-vivo studies had demonstrated that density and infectivity of virus were enhanced by the co-infected bacterial reaction [22, 23].

All 11 cases of co-infection of both dengue and leptospirosis were confirmed using laboratory test. The methods used were real-time PCR and NS1 antigen detection for dengue virus while IgM latex agglutination and microscopic agglutination test (MAT) were used to identify leptospira. A positive IgM assay with a positive (titer ≥ 400) or negative (titer < 50) MAT indicate current infection of leptospirosis whereas a negative IgM with positive MAT indicates past infection. Also, MAT is considered as the gold standard test for diagnosis of leptospirosis. However, although MAT is a high specificity test that detects the total immunoglobulin, it often shows false negative results reflecting low sensitivity compared to IgM ELISA / latex agglutination test [24]. A strong positive results indicated by the four-fold rise in titer between the acute and convalescent phase samples or seroconversion. In our study, six out of the eleven IgM positive leptospirosis cases had inconclusive result for MAT with titer ranged between 50 to <400. According to the original Faine’s criteria (WHO guideline), a MAT titer of >100 can be considered as positive in low endemicity region, whereas a titer of ≥400 was the cut-off implied in the high endemicity region [25]. However this criteria was excluded in the modified Faine’s criteria as it further complicated the final diagnosis of leptospirosis [26]. Despite that, the data analysis herein revealed a positive IgM and inconclusive MAT, which is because of the representative of early antibody in the current infection with presence of residual antibody from a past infection. This can be justified on the ground that over time, the antibody levels gradually drop following the infection, although low titers may persist up to 10 years of post-infection. In a previous study conducted to validate the microsphere immunoassay and MAT for detection of leptospirosis, it was found that the microsphere assay is actually reactive towards the samples with low titers for MAT [27]. Therefore, it can be confirmed that concurrent serological assays are required along MAT for confirmative diagnosis of leptospirosis.

In an attempt to identify the predictors for dengue-leptospirosis co-infection, some comparative analyses are conducted with demographic, clinical, and laboratory parameters between dengue-leptospirosis and non-leptospirosis groups. Chi-square analysis showed significant correlation between gender and dengue-leptospirosis, where male patients are more susceptible to co-infection compared to female patients. Although the association of gender for dengue-leptospirosis co-infection was investigated before, but studies revealed that male patients had higher risk of dengue and leptospirosis infections when these diseases are present independently [28, 29]. In Malaysia, summary findings of literatures from the year 1964 -2016 revealed that the ratio of male infected with only leptospirosis ranged from 1.9 - 4.3 as compared to female [30–34]. Meanwhile, our study not only showed similar trend but male had much higher risk than female in acquiring co-infection of leptospirosis and dengue (10:1). Occupational exposure could be one of the reasons for male preponderance. Construction sites, sewage area, rice field, and sugar cane farms generally consisted of more male workers, where these are the locations that often deemed for acquiring dengue and leptospirosis infections. In the present study, the multiple logistic regression reached borderline significance for gender which can be due to the sample size.

In this study, a wide range of clinical symptoms were investigated for their association with dengue-leptospirosis co-infection. Our investigation showed that patients with the co-infection were significantly present with shock. However, there was only patient with shock symptom in the co-infected group. This patient was referred to the hospital as a case of dengue fever with compensated shock and sinus tachycardia. Further laboratory result revealed that the patient developed warning sign of hemoconcentration and had underlying diseases which are diabetes and hypercholesterolemia. However patient eventually recovered from shock after treatment. Other laboratory inspection revealed positive result for dengue IgM, NS1 antigen, leptospirosis IgM and inconclusive MAT (titer 1: 200). Platelet count and CPK level were normal. In this case, CPK level could not predict leptospirosis. Although statistically significant, we believe that shock as a predictive factor for dengue-leptospirosis co-infection need be interpreted cautiously.

Symptoms including headache, arthralgia, epigastric pain, retro-orbital pain, gum bleeding, and bleeding tendency were exclusively present for dengue fever cases without leptospirosis. Therefore, these symptoms are clear indication for the differentiation of dengue from leptospirosis. A study had revealed higher correlation of abdominal pain during leptospirosis infection, while arthralgia during dengue infection [35]. However, in our study, only one patient from the co-infected group was present with abdominal pain, therefore statistically insignificant. In addition, no fatality was documented in all 11 dengue-leptospirosis co-infected patients. These cases were also diagnosed as classical dengue, on the other hand, three severe dengue cases were found in the dengue infection without leptospirosis group. This data suggests positive outcome on the survival of co-infected patients provided there is fast and accurate detection of both pathogens and prompt treatment of patients.

As part of the study, the laboratory parameters did not reveal any significant value as co-infection predictor. Nevertheless, a 100% frequency of DENV 1 can be detected among the patients that were infected with both dengue and leptospirosis. Thus, this finding raised the question that if mosquitoes carrying a certain dengue serotype selectively target patients with leptospirosis. However, since DENV 1 is found at high frequency in our study and could have elevated the chances of co-infections with this serotype, thus it is difficult to draw conclusion of serotype prevalence at this point. There is extremely limited literatures available to correlate dengue serotype with concomitant leptospirosis and dengue infection cases. Only one study has reported on the prevalence of dengue serotype of the patients with leptospirosis co-infection which was found to be DENV 2 [17]. Similarly, this result is inconclusive, as the predominant serotype circulating during the period of the study was DENV 2. In spite of that, since not many studies have emphasized and documented the serotype of dengue virus among leptospirosis co-infected patient, we hope that our findings will trigger interests on further studies related to this.

Another laboratory parameter that ought to be taken into consideration in an event of dengue-leptospirosis co-infection is CPK level. CPK has been associated as a predictor for leptospirosis [36]. The present study investigated the significance of CPK in dengue-leptospirosis co-infected cases. Despite that the CPK level was not significant, the mean CPK in the dengue-leptospirosis co-infection group is substantially higher (almost tripple) as compared to the dengue infected group. The insignificance could be due to small number of co-infected patients. CPK is commonly raised in patients with leptospirosis due to myalgia. Additionally myalgia is also a prominent symptom in dengue infected patients. Therefore it is postulated that CPK level in dengue-leptospirosis co-infected patients is expected to be raised higher than dengue infected patient as a result of extreme myalgia, supported by our findings.

Our current study has several strengths and limitations. We investigated the prevalence of concurrent dengue and leptospirosis infections among confirmed dengue patients and found a total of 11 cases (4.1%) while many other studies have reported only one to three individual cases or much lower prevalence compared to our study [6, 8, 13, 15, 16, 18, 20, 21, 37]. In addition, our findings may warrant further study to elucidate the occurrence of concurrent dengue and leptospirosis infection. Among the limitations of the current study is lack of robust tool to detect dengue and leptospirosis simultaneously, nevertheless it can be developed in future. Our current study has focused on one locality only, therefore we suggest future studies in Malaysia involving bigger study area to draw strong evidence on occurrence of dengue and leptospirosis co-infection and the clinical predictors.

## Conclusion

In conclusion, patients with dengue fever who suffered from shock were more likely to be affected from co-infection compared to patients that did not experience shock, however this findings need to be treated cautiously due to the low number of patients presented with shock in the current study. Male patients and those who are infected with DENV 1 might need close monitoring for co-infection with leptospirosis as there is possibility for association of gender and dengue serotype with co-infection. Every possible method is required to enable the identification of the patients that were at risk of being co-infected by dengue-leptospirosis. The identification will lead to further intervention to possibly be instituted.

## Abbreviations

ALT: Alanine transaminase
AST: Aspartate transaminase
CI: Confidence interval
CPK: Creatine phosphokinase
Cy5: Indocarbocyanine 5
DENV 1: Dengue virus serotype 1
DENV 2: Dengue virus serotype 2
DENV 3: Dengue virus serotype 3
DENV 4: Dengue virus serotype 4
ELISA: Enzyme linked immunosorbent assay
FAM: 6-carboxyfluorescein
HEX: Hexachloro-6-carboxyfluorescein
IBM: International business machines
IgG: Immunoglobulin G
IgM: Immunoglubulin M
MAT: Microscopic agglutination test
NS1: Non-structural 1
PCR: Polymerase chain reaction
RNA: Ribonucleic acid
RT: Reverse transcriptase
SPSS: Statistical package for the social sciences
WHO: World Health Organization
